# Facilitators and barriers to chronic non-communicable disease management under family doctor contracting services in China

**DOI:** 10.3389/fmed.2025.1506016

**Published:** 2025-03-11

**Authors:** Rui Jiang, Yuze Xin, Shuangjie Peng, Yuhan Zhou, Xinyi Zhang, Yu Shi, Guangming Chang, Min Yang, Lvzhuang Huang, Lingling Xu, Xinrui Wei, Yongchen Wang

**Affiliations:** ^1^Department of General Practice, The Second Affiliated Hospital of Harbin Medical University, Harbin, China; ^2^School of Public Health, Harbin Medical University, Harbin, China; ^3^Department of Health Management, School of Public Health, Tianjin Medical University, Tianjin, China; ^4^Department of Medical Ethics, The Second Affiliated Hospital of Harbin Medical University, Harbin, China

**Keywords:** non-communicable diseases, family doctor contracting service, RE-AIM framework, qualitative study, primary health care

## Abstract

**Background:**

Chronic non-communicable diseases (NCDs) pose a significant health burden in China exacerbated by population aging and rapid urbanization. The Family Doctor Contracting Service has been implemented in China as a primary health care approach to improve NCD management and overall health outcomes. This study aims to identify factors associated with implementing chronic NCD management under the FDCS in the Chinese primary health care system.

**Methods:**

This qualitative study was conducted in 4 purposively selected cities in China. Health administrators from the local health commission, staff members from local primary health care facilities, and community-dwelling individuals with NCDs were recruited using purposive and snowball sampling. The reach, effectiveness, adoption, implementation, and maintenance (RE-AIM) framework was adopted to inform our interview guides and data collection and analysis. Themes regarding barriers and facilitators were generated using deductive and inductive approaches.

**Results:**

A total of 140 participants were interviewed 82 (58.6%) were female and the mean (SD) age was 51.0 (13.68) years. Significant barriers included low health literacy levels, limited awareness about NCD, insufficient healthcare professionals and medical resources, poor publicity and regulation, limited multisectoral collaboration, and inadequate audit and feedback systems. Facilitators included affordable and convenient primary health services, recognition of the indispensable benefits of NCD management, good patient-physician bonds, and the high priority given by local governments.

**Conclusion:**

This qualitative study identified significant facilitators and barriers to the implementation of NCD management under the FDCS at the primary care level. These insights can contribute to better NCD prevention and management implementation in the Chinese primary health care system.

## Introduction

The global epidemic of chronic non-communicable diseases (NCDs) has imposed a heavy disease burden on the general public. According to the World Health Organization (WHO), deaths from NCDs are expected to reach 55 million by 2030 (1). The high and increasing prevalence of NCDs is a significant health problem and a formidable challenge for China (2–4). The Global Burden of Disease Study(GBD) 2019 revealed that NCDs accounted for 84.93% of the total disease burden in China (5). Strong political will is evident in addressing primary health care in China through policies and increased health expenditures aimed at strengthening the primary health care system to tackle the dual burden of NCDs since 2009 (6, 7).

The Family Physician Program (FPP) proposed by WHO is a health strategy to manage NCDs. To deliver primary healthcare, family physicians are crucial in diagnosing, managing, and treating hypertension and diabetes as NCDs. Based on the experiences of several countries, Family Physicians are fundamental for quality improvement, cost-effectiveness, and equity in health care systems (8, 9). China first launched the Family Doctor Contracting Service (FDCS) program as an innovative and fundamental health system reform policy in 2009, officially implementing it nationwide in 2016 to provide primary health care and improve the community’s awareness of NCDs (10, 11). The National Essential Public Health Service (NEPHS) project mandated primary care facilities to provide services to residents through the use of FDCS. China’s FDCS relies on a team of family doctors, including family doctors, community nurses, public health physicians, etc. Their responsibilities include creating health records for residents, diagnosing and treating common diseases, preventing and managing NCDs such as hypertension and diabetes, and making referrals by appointment.

The FDCS program has made remarkable progress in aims to improve access to primary health care services and prevent NCDs such as hypertension and diabetes mellitus. Patients who suffer from NCDs, particularly the older adult, are more likely to accept and use FDCSs to meet their health needs, and willingness to sign contracts increases with the severity of NCDs (12, 13). However, there is lower willingness among the general population to contract with family doctors (14), and distrust in the quality of NCD management under the family doctor (15). Unfortunately, existing studies mainly focus on residents’ willingness to contract with family doctors and limited investigation of the FDCS’s effect on NCD prevention and management. To remedy this gap, this study aims to explore stakeholders’ perceptions of facilitators and barriers in implementing NCD management under the FDCS in China. Additionally, it offers recommendations for better implementation of the management of NCD under the FDCS.

## Methods

### Study design and sites

This qualitative study employed face-to-face, in-depth interviews, and focus group discussions to collect data with the help of an interview Guide. The study required an in-depth understanding of stakeholders’ views on NCD management under the FDCS. Focus group discussions provide rich data on social norms, feelings, attitudes, and perceptions (16). The study followed the Consolidated Criteria for Reporting Qualitative Research (COREQ) (17).

**Figure 1:**
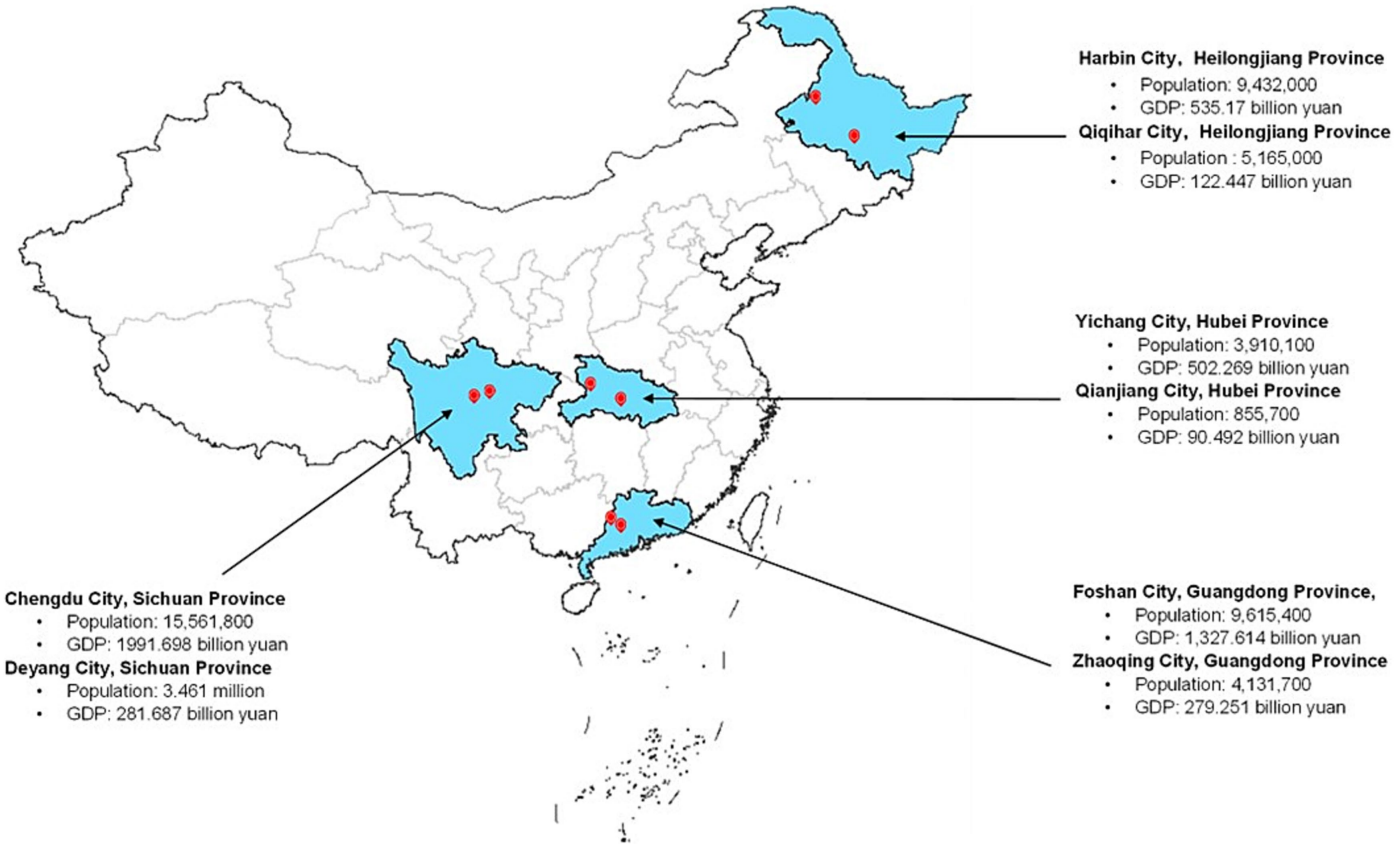
Geographic location.

This qualitative study was conducted in 4 purposively selected study sites with diverse geographic locations and socioeconomic status (18–21): Harbin and Qiqihar City from Heilongjiang Province, Chengdu and Deyang City from Sichuan Province, Yichang and Qianjiang City from Hubei Province, and Foshan and Zhaoqing City from Guangdong Province (Figure 1; Supplementary file S1). The Medical Ethics Committee of the Second Affiliated Hospital of Harbin Medical University approved the study (KY2020-091).

### Study participants and sampling

Study participants were recruited from three stakeholder groups, including representatives of Policymakers, health care providers, and NCD patients. Policymakers from provincial and municipal health commissions and health care providers, as well as health administrators and staff members from primary health institutions, were eligible if they had worked more than 1 year in their organizations, were responsible for or provided technical support for NCD management in the FDCS, and were willing to sign informed consent. Community-dwelling individuals diagnosed with NCD (i.e., hypertension and/or Type 2 Diabetes) aged 35 years or older were eligible if they lived in the community under the jurisdiction of selected primary health care institutions and were willing to provide informed consent. Those with severe medical conditions unsuitable for interviews were excluded. Before conducting interviews and discussions, participants provided written informed consent, including permission to be audio recorded. A combination of purposive and snowball sampling was used to recruit participants.

### Data collection

Semi-structured interview guides were created based on the reach, effectiveness, adoption, implementation, and maintenance (RE-AIM) framework domains. The RE-AIM framework has been widely used in qualitative and quantitative components to plan and evaluate health programs and policies (22, 23). Example questions for assessing each dimension are shown in Table 1. The interview guides (Supplementary file S1) were adapted to the different roles of the stakeholders.

**Table 1:** Reach, effectiveness, adoption, implementation, and maintenance (RE-AIM) framework dimension and example interview questions.

Dimension	Example interview questions
Reach	Do you think that the existing measures for NCD management can meet the actual demand for health care services? What is the coverage of these policies or measures? How can higher population coverage be ensured?
Effectiveness	What do you think about the evolution of existing policies related to NCD management from central to local level? What factors do you think have contributed to the regional differences in the policy evolution? How effective have these policies been in their implementation? What factors influence the effectiveness of these policies? How does your primary health care organization evaluate and measure the effectiveness of its policies?
Adoption	How is the local uptake of measures related to NCD management? How does your department ensure that these policies are well accepted and adopted by health service providers and NCD patient and their families?
Implementation	Overall, how do you perceive the process of implementing measures related to NCD management on the ground? What are the facilitating and hindering factors? Is your current work related to NCD management in any way? What is the reality of the implementation process of these policies? What factors affect the reality and quality of policy implementation?
Maintenance	Do you think the existing interventions related to NCD management under the contracted family doctor service model are sustainable? How do you ensure the sustainability of these policies or interventions with long-term impact? What are the means of long-term performance evaluation?

All interviews were conducted by a qualitative researcher (RJ, female with clinical or public health backgrounds) and two notetakers (YZ and MY, both female master’s students), to ensure privacy and facilitate the free sharing of views. All individuals involved in the study received training to ensure that they were aware of (1) the importance of confidentiality, privacy, and the ethical considerations associated with data collection, (2) the need to avoid pre-assuming the attitudes of the participants, and (3) the obligation to maintain a neutral stance and not express judgment for the views of the participants. The researchers and participants were not acquainted before the interview, and no repeat interviews were conducted. Interviews were conducted in a quiet and private space to ensure confidentiality and freedom to share views without external influence. Recruitment of participants ceased upon reaching thematic saturation, indicating that no new responses were being identified. The interviews were transcribed anonymously, with sound recordings and transcripts securely saved on a password-protected personal computer.

### Data analysis

Interviews were audio recorded and transcribed verbatim. Participants were interviewed solely in Mandarin Chinese, with quotations translated into English via forward translation (from the source language into English) and back-translation (from English to their source language). Thematic analysis was used to develop a descriptive account of participants’ perceptions and interpretations. The RE-AIM framework guided the initial analysis (deductive approach), and thematic analysis identify codes to related to each dimension within the framework (inductive approach). First, two researchers (RJ, YX) independently reviewed transcripts and generated preliminary codes about influencing factors around emergent concepts. Second, similar preliminary codes were grouped into themes and deductively mapped to the RE-AIM framework. A coding framework was generated using constant comparison to establish a hierarchy of conceptual codes until no new themes were identified. The codes (RJ, YX, and SP) were refined through iterative discussion, coding, review, and re-coding over several meetings. Finally, common barriers and facilitators were further grouped according to the five domains, and common context-specific themes influencing NCD management provision were identified. Each cited quotation was marked by participant role and their study number to avoid identifiable information. Data coding and analysis were conducted in Chinese using the NVivo analytical software system, version 12 (QSR International).

## Results

There were 84 in-depth interviews (30 to 60 min for each) and 11 focus group discussions (about 4 participants in each session lasting for 60 min) completed, with a total of 140 participants (58.6% female, mean age 51 ± 13.68 years). 73.3% of service professionals have worked for more than 10 years in their positions, and 75.6% have a college degree or above. The interviews were conducted between December 2023 and March 2024. Demographic information and categorical results are summarized in Table 2. Facilitators and barriers are listed in Table 3 based on the RE-AIM domains. Findings were aggregated for each RE-AIM dimension around barriers and facilitators.

**Table 2:** Characteristics of participants.

Demographic characteristic	In-depth interview			Focus group discussion	
	Policy makers (*n* = 14)	Leaders of primary health institutions (*n* = 20)	Healthcare providers (*n* = 52)	Patients with chronic diseases (*n* = 54)	Total (*n* = 140)
Gender					
Male	9 (64.3)	10 (50.0)	15 (28.8)	24 (44.4)	58 (41.4)
Female	5 (35.7)	10 (50.0)	37 (71.2)	30 (55.6)	82 (58.6)
Age, mean(SD), y	41.4 (3.82)	46.4 (6.21)	40.6 (7.94)	65.2 (8.24)	51.0 (13.68)
Education					
College and above	14 (100)	15 (75.0)	36 (69.2)	NA	65 (75.6)
High school and middle school	0	5 (25.0)	16 (30.8)		21 (24.4)
Primary school or below	0	0	0		0
Years of working					
5 y or below	0	1 (5.0)	9 (17.3)	NA	10 (11.6)
5y to 10y	5 (35.7)	1 (5.0)	7 (13.5)		13 (15.1)
10y and above	9 (64.3)	18 (90.0)	36 (69.2)		63 (73.3)
Years of NCD					
5 y or below	NA	NA	NA	20 (37.0)	20 (37.0)
5y to 10y				8 (14.8)	8 (14.8)
10y and above				26 (48.1)	26 (48.1)
Location	14	19	53	54	
Heilongjiang	3	8	14	24	
Sichuan	3	4	14	12	
Hubei	4	3	16	10	
Guangdong	4	4	9	8	

NA, not applicable.

**Table 3:** Identified themes, and barriers to, and facilitators under RE-AIM.

Domain	Facilitators (10)	Barriers (12)
Reach	Primary healthcare services provide convenient services, preferential prices and high reimbursement ratios	Limited NCD management services Differences in population mobility in different economic development regions Low awareness of health management among patients, especially those aged 35–64 with low rates of knowledge and acceptance
Effectiveness	Both doctors and patients agree that health situations have improved Strict quality control and performance appraisal of health records	Insufficient infrastructure and medicines in primary healthcare facilities Limited and small-scale health promotion activities
Adoption	Doctors and patients generally agree that accepting chronic disease management services from family doctors is necessary Regular community health promotion and high-risk population management Local governments in affluent regions prioritize and allocate significant resources to NCD management.	Inadequate publicity and regulation of policies by the government and media Lack of guidance and incentives (performance appraisal, talent development)
Implementation	Personalized health management programs Medical personnel establish good doctor-patient relationships Collaboration with community boards and grid workers The health administration department guides primary medical institutions and organizes training for medical personnel.	Insufficient number of medical staff and insufficient capacity in primary health care facilities (including general practitioners and public health personnel) Lack of collaborative support beyond health administration Health system service information management needs improvement
Maintenance	–	Lack of multi-dimensional assessment and evaluation indicators Poor salaries and high mobility of medical personnel

### Reach

All participants were highly satisfied with the coverage achieved of primary health care services provided by the family doctor. The affordability and convenience of primary health care and a high proportion of medical insurance reimbursement are the main facilitators of FDCS reaching a wider population. The majority of participants had a positive view of FDCS, believing that the goal of promoting FDCS was to reach the whole population, not just hypertensive or Type 2 Diabetes patients.

*“The community health center is just a few minutes’ walk away from home. Prescriptions of medicines and tests are all very cheap, and my family doctor regularly calls me for health check-ups, which are free of charge. It is convenient for the older adult, especially us!”* (Patient 50, Guangdong Province).

Health care providers recognized the value and benefits of NCD management in improving health but noted limited medical services and could not meet patients’ actual medical needs.

*“Patients are reluctant to come because they think that they cannot do any check-ups and laboratory tests in the community health center, and their problem cannot be solved. It would be better to go directly to a higher hospital for treatment.”* (Health Administrator 20, Sichuan Province).

Most policymakers and health care providers cited low health literacy and limited NCD awareness as barriers. Particularly, patients aged 35–64 with NCDs had lower awareness and acceptance, owing to most of these people being employees and not cooperating with the medical services provided by the family doctor team on working days.

*“Patients’ compliance is poor, health awareness is low, especially for chronic disease patients over 65 years old who think they are still young and in good health, there is no symptom and they do not need the management of the family doctor; especially among these people who are still working, it causes resentment if you always call them.”* (Staff members 38, Hubei Province).

Participants also highlighted that economically developed areas have a large labor force, but they also experience significant population mobility, making it challenging for family doctors to provide contracted services. Meanwhile, in economically disadvantaged regions, such as certain provinces in Northeast China, there is a growing issue with population outflow and an increasingly aging population. These areas have unique cultural practices, such as “mao dong” (staying at home and minimizing outdoor activities in winter) and “nan fei” (older adult migrating south for winter) make regular primary medical facility visits difficult.

### Effectiveness

The high effectiveness of FDSC in preventing and managing NCDs includes a high degree of recognition between doctors and patients, strict quality control and performance appraisal that were identified as facilitators. Both healthcare providers and NCD patients described significant health condition improvement with FDSC services. Healthcare providers noted effective management of blood pressure and blood sugar levels, improving care satisfaction and increasing compliance. The ongoing evaluation of residents’ health records demonstrates the health administration’s commitment to providing quality healthcare.

*“The province conducts a comprehensive assessment of service management every year, and quality control is regularly conducted within facilities to review residents’ health records rigorously.”* (Policy marker 6, Sichuan Province).

Significant barriers included insufficient infrastructure and medicines, weak health education and promotion. All interviewees identified timely updates and effective articulation in the National Reimbursement Drug List (NRDL) as a preferred strategy. The current non-pharmacological interventions were limited to health promotion (e.g., organizing expert lectures, distributing educational materials, and broadcasting videos) for NCD patients and high-risk populations.

*“The* var*iety of medicines is not sufficient, some medicines are only available in secondary and tertiary hospitals and cannot be purchased in our primary health care facility. So patients will not want to come back later if they cannot get a prescription from you.”* (Staff members 20, Sichuan Province).

*“Community health centers do too few tests, the equipment is not as good as the higher hospitals, and many imported drugs are not available here.”* (Patient 31, Hubei Province).

### Adoption

Key factors that significantly impact the adoption include the necessity of FDCS, regular health education activities, and high financial investment. Most interviewers identified that FDCS at the community level plays a crucial role in implementing NCD management strategies and programs. Increasing government attention to the growing burden of NCDs, aligned with the goal of Healthy China 2030, has led to a stronger resident engagement with family doctors for NCD prevention and management services. Many policymakers mentioned that Healthy China 2030, one of the most important initiatives of the Chinese government, provided an opportunity for health education and promotion to high-risk populations. In addition, local governments in economically developed areas attach great importance to increased financial investment, which is conducive to providing more and better services, not only for patients with hypertension and diabetes but also for other NCDs.

*“There are too many patients with diabetes and hypertension in our country, and complications are very common. Family doctor contracted service is currently the most efficient means to prevent and manage chronic diseases. We regularly conduct health promotion activities in the community, measure blood pressure and blood glucose, and provide dietary and exercise guidance to residents, who are generally very willing to accept the family doctor contracted service.”* (Staff members 5, Heilongjiang Province).

*“The local government attaches importance to it with much financial investment. Based on good preventive management of the two diseases, it will carry out some self-selected programs such as the preventive management of cardiovascular diseases and chronic obstructive pulmonary disease. The quality of the equipment in the community health center has also been improved*.” (Policy marker 11, Guangdong Province).

The primary reason for hindered residents’ adoption of the FDCS was a lack of understanding of relevant policies about the FDCS program and hierarchical care. Therefore, they did not initially seek medical treatment from family doctors at primary care institutions; instead, they tended to go to high-level hospitals, which is a common habit among residents in China. The interviewers suggested that the government and state news media should collaborate to expand the publicity and coverage, prioritizing promoting and popularizing FDCS, hierarchical care, and basic public health services among residents. Mass media campaigns by the government were suggested as a critical channel to disseminate information to enhance FDCS use effectively.

*“I’m not really sure what hierarchical care is all about, and I do not have a referral to a higher hospital through my family doctor.”* (Patient 19, Heilongjiang Province).

*“The health insurance policy for chronic diseases is not yet perfect, and in many areas, the identification of chronic diseases still requires a visit to a higher hospital.”* (Health Administrator 12, Sichuan Province).

*“The community is full of domestically produced medicines. I do not know what the difference is with the imported ones, and a lot of the medicines to improve circulation are out of pocket. My family doctor gave me a lot of advice, but I’d like to hear an official report from the government.”* (Patient 45, Hubie Province).

Health care providers highlighted three major challenges: (1) The proliferation of false medical information such as new media and short videos; (2) Residents’ inability generally to identify false medical information; (3) Lack of government department supervision.

Financial incentive mechanisms are necessary to enhance family doctors’ motivation, however, there are no effective incentives in place for family doctors as a barrier. The increased workload for family doctors in primary health organizations has not been matched with additional payments beyond their original salary. Although local governments have implemented non-financial incentives such as title promotion, domestic training opportunities, and honorary rewards, there are few incentives and weak influence of performance-based salary. As a result, family doctors may have a lower sense of achievement and a preference for outpatient services.

*“It can be said that the work of the family doctor is not proportional to the pay and reward. Some performance tilt the organization to give health care workers, but more incentives still need to be supported by guidelines and rules. After all, we go through a rigorous annual audit every year.”* (Health Administrator 16, Hubei Province).

*“The part of public health management is inherently unattractive and unfulfilling, and GPs are more willing to engage in primary care services.”* (Health Administrator 6, Heilongjiang Province).

### Implementation

Personalized care approaches, good patient-physician bonds, close collaboration with community groups, strengthened guidance and support from health administrative departments, and active training for various medical personnel were identified as potential implementation strategies for maintenance and sustainment. All patients strongly desired maintain long-term contact with their family doctors appreciating their responsibility, service attitude, and patient-centered approach. Likewise, the family doctor maintains good working relationships with community neighborhood committee and the grid staff. From the perspective of policymakers, we take responsibility for strengthening primary health care service capacity through various types of training activities.

*“My family doctor is very nice and will stand in my point of view for me. We can contact each other at any time by phone or WeChat, most of my problems are solved by my family doctor.”* (Patient 25, Sichuan Province).

*“We have a very close relationship with the community councils and grid workers. Due to our close cooperation, we have been able to carry out health education in the community regularly and successfully. During the three-year COVID-19 epidemic, we worked more closely with grid workers and residents, collecting nucleic acids, which increased residents’ trust in us.”* (Health Administrator 4, Heilongjiang Province).

*“Provincial and municipal health administrations have strengthened their work responsibilities regularly organize and carry out* var*ious types of medical training covering public health and basic medical care and so on. The strong medical services capacity is the basis for developing chronic disease management, meeting residents’ diverse medical needs.”* (Policy marker 10, Hubei Province).

Key factors influencing NCD management implementation under FDCS include shortage and insufficient service capacity, heavy workload, inadequate health system service information system, and limited multisectoral collaboration. Health care providers reflected that heavy workloads and staff shortages constrained capacity building. Of note is that the shortage of staff is not only reflected in family doctors but also in the case of public health professionals, as there is difficulty in implementing NCD management. Although digitalizing health systems was considered a national policy priority, especially in economically backward areas, the fragmented and siloed information systems still need to be addressed. The policymakers highlighted the importance. Quality-oriented performance evaluation could be considered in the ongoing process of FDCS to strengthen multi-sectorial collaborations.

*“Inadequate capacity of primary health care providers, insufficient manpower, heavy workload, has been longstanding problems. We have tried* var*ious solutions, such as improving the informatization of public health services the opening green channels for timely patient transfers to higher hospitals, but there is still a large gap from the ideal goal.”* (Policy marker 4, Sichuan Province).

*“Nowadays, the construction of information technology systems is underway, but frequent company changes have caused port connection issues, resulting in information management obstacles, loss of information, and time-consuming data entry.”* (Staff member 2, Heilongjiang Province).

*“Implementing the family doctor contracted program requires multi-departmental collaboration, not just one or two departments. It needs high-level provincial government attention, financial support, and many other aspects.”* (Policy marker 14, Guangdong Province).

### Maintenance

The main barriers to the maintenance development of NCD management FDCS include the need for multi-dimensional, scientific, and flexible assessment indicators and the poor remuneration and high turnover of medical staff in primary medical institutions. There are concerns that the current evaluation feedback system does not accurately reflect the health situation of NCD patients. Additionally, it is time-consuming for medical staff to implement. Health administrators have stressed that the staffing levels have not been met and the salary package is not attractive, resulting in a significant loss of medical staff. This makes it difficult to attract and retain excellent personnel.

*“At present, there is a lack of scientific and flexible assessment and evaluation indexes for the health records we manage. The national indexes issued to localities should be based on the actual level of economic development, the characteristics of the disease spectrum of the population, geographical area climate, and other multi-factors that affect the comprehensive consideration of the formulation of the indexes. The assessment should be focused on quality management.”* (Health Administrator 6, Heilongjiang Province).

*“Working in primary health care facilities is often not the first choice of many people, some people do it for no night duty or proximity to home for family care. But when work is too heavy, the treatment can not be improved, staff establishments can not be implemented, some people choose to leave. So mobility of primary care providers is higher than large hospitals.”* (Staff members 38, Sichuan Province).

## Discussion

This qualitative study involved multiple stakeholders to understand the process, challenges, and recommendations associated with NCD prevention and management under FDCS in China. The significant facilitators were affordable and convenient primary health services, recognition of the indispensable benefits of NCD management, good patient-physician bonds, and the high priority given by local governments. In contrast, the major barriers included low health literacy levels, limited awareness about NCD, insufficient healthcare professionals and medical resources, poor publicity and regulation, limited multisectoral collaboration, and inadequate audit and feedback systems. Understanding implementation barriers from a theoretical perspective is crucial for identifying solutions to overcome them. Based on RE-AIM, this study proposed context-specific implementation strategies that may be suitable for systematically addressing barriers to NCD management under FDSC in China.

Generally, primary health care can effectively reduce the morbidity and mortality of NCD, manage patient conditions, and reduce the burden of NCD (24, 25). The family doctor system represents a significant initiative aimed at enhancing the quality of primary health care services, has been implemented in over 50 countries and regions, and has achieved gratifying results in various respects, which has garnered the attention of governments and medical communities (26). The United Kingdom required residents to register with general practitioners when the National Health Service was established in 1948, while the United States implemented Medicare and Medicaid in the 1960s (27, 28). Residents in Germany are free to choose their general practitioners for contracted services (29). Different national conditions lead to variations in the specific service modes and operational mechanisms within the family doctor service system. In a previous study conducted in Canada, it was found that family doctor system improved the management of hypertension (30). International evidence supports the necessity of FDCS implementation in developing countries. A systematic review from Turkey discovered a significant improvement in hypertension awareness, treatment, and control rates from 2003 to 2012 (31). Iran has decided to extend the family physician program to all provinces in 2022 and 2023 because of improving raised people’s awareness of NCD and encouraged lifestyle changes (32).

In China, poor publicity and regulation, limited health education and promotion services, and low health literacy levels compromise the understanding of NCD prevention and management. In addition, a significant disparity in awareness of NCDs was also found across different socioeconomic development regions. Patients with higher levels of perceived health literacy have higher levels of engagement in health promotion behaviors, consistent with previous studies (33, 34). Of note, NCD patients are not free from age-related stereotypes when they are faced with health problems and tend to believe that the decline in their physical functions is a natural aging process, so prevention and management are unnecessary. This is a reminder that policymakers and healthcare providers should consider education, income, and urban and rural areas when formulating policies and prevention plans for health promotion. Our research indicates that the government could enhance public health promotion through promotional materials, media campaigns, and billboards. Moreover, NCD-related health education and promotion services should be provided by family doctors during outpatient and further penetrate residential areas to carry out more diverse health education to public information campaigns. Although social media hosts opportunities for residents to exchange health messages on popular social media channels, including WeChat, TikTok, and Red Booklet, through various modalities (e.g., text, image, video, and gif), the authorities must strictly supervise the misleading medical information.

Adequate availability and distribution of healthcare staff members, especially family doctors, are crucial to increasing access to essential health services and achieving the desired NCD management outcomes (35). The current policy strategies focus on recruiting more medical students for rural areas, providing community-based medical education, requiring mandatory service, and placing doctors in their hometowns to improve retention. Meanwhile, a residency training program (also known as the ‘3-year general practice standardized training’) was initiated in 2011 to attract new qualified physicians to become family doctors in primary health institutions (36, 37). The program aims to provide high-quality training to meet the public’s increasingly high expectations for health care services. Despite government efforts in incentives, recruitment, training, and retention, healthcare professional shortages persist. Our study revealed considerable discrepancies in the financial and non-financial incentives among different areas, including policies on increasing income level, supporting medical education, and providing more professional career development opportunities, which greatly influenced the performance of family doctors. Only when the normalized incentive mechanism is established nationally can the family doctors’ contracting service be sustainable. We also argue that nurses and rehabilitation professionals are a neglected human resource and could be task-shifted to supplement family doctors for NCD prevention and management.

China began to emphasize FDCSs in 2009 to promote a hierarchical diagnosis and treatment system and encourage the residents first to visit primary care facilities (38). Challenges related to coordination between different healthcare levels and specialized hospitals resulted in fragmented health care and reduced patient NCD satisfaction. Although China has made efforts to improve the infrastructure, expand the range of medicines, and widen the disparity in medical insurance compensation levels in primary care institutions, there remains a considerable gap between community healthcare institutions and hospitals. China has achieved universal health insurance coverage, but most schemes prioritize inpatient care instead of outpatient care, which has identified substantial costs incurred from frequent visits for NCD treatments (39). Policymakers and health administrators are advocating for a multi-departmental joint promulgation of strategies to promote hierarchical diagnosis and treatment including updating the drug catalog in primary medical institutions, establishing a generalist-specialist combination management model, and information interconnection between medical institutions. Based on the experience shared in previous studies (40), a ‘green channel’ could be established in the hospital, making it possible for NCD patients to receive early screening and treatment, obtain effective and convenient follow-up and referral services, and improve their quality of life. Patients with complications could access treatments with better precision. In economically developed areas, local governments prioritize NCD management and invest significant financial resources in building informatization system construction and providing comprehensive and ongoing training for medical personnel, furthermore, efficient informatization and intra-regional information connectivity have greatly improved the development of NCD prevention and management under FDCS.

Periodic feedback assessments and efficient audits are essential for continual quality improvement in managing NCDs (41, 42). However, the absence of performance indicators in the current audit and feedback loop could impact the quality of the implementation and health outcomes. A key finding in our research is that quality improvement or performance measurement in NCD management using purely biomedical indicators or indicators based on adherence to guidelines from the government may lead to a loss of care quality and the ability to measure equity in health care. Normative needs are defined by policymakers and health care professionals (e.g., family doctors, public health workers, and nurse practitioners) and felt need, asking patients what they feel should be taken into account in trying to attain health equity (43). Our results show that process-oriented indicators could gage the individual effort of family doctors, such as the number of follow-ups per year (whether face-to-face or telephone), the number of health promotion activities, and the degree of satisfaction of patients toward the current management. There exist various complex factors affecting the NCD situation, and health providers recommend that more experienced clinical specialists and general practitioners should participate in the assessment and feedback, to ensure the flexibility and scientific nature of the assessment indicators and tailor the intervention to the local context.

### Strengths and limitations

Several facilitators and barriers emerged from our research, shedding light on the complexities of implementing chronic NCD management under the FDCS in China. This study’s strengths include the depth of data collection, the use of an established implementation science framework, and the collection of perspectives from multiple key stakeholders, including representatives of policymakers, leaders of primary health institutions, family doctors, and patients with NCD from primary health institutions with different economic developments and geographic locations. The RE-AIM framework helped semi-structured interviews to examine the feasibility and facilitators of implementing chronic NCD management under the FDCS in different contexts. The current study has some limitations. First, data were collected from key informants in only four provinces, which may limit the generalizability of the study findings, although representation did include the Central, East, West, and North regions. Second, all factors were identified qualitatively without establishing causal relationships. Further quantitative surveys are recommended to validate these findings.

## Conclusion

Based on stakeholders’ perspectives and experiences, the FDCS program has been implemented smoothly in the current context. At the same time, the FDCS has faced multiple challenges in implementing NCD prevention and management. The participants highlighted that health education and health promotion may increase the awareness of NCD management and improve health literacy. More policy strategies could focus on strengthening medical publicity and supervising, as well as continuous investment in reinforcing the implementation of strengthening the FDCS program and improving hierarchical care. We recommend further facilitating and supporting multi-sectorial collaborations in policy-making and execution processes. Future investigations should be established to audit and feedback systems. This study found multilevel challenges attributed to NCD prevention and management. It reinforced the implementation of strengthening the FDCS program to provide an enabling and supportive environment for integrated primary health care. The current era’s volatility, uncertainty, and complexity present a new framework for China’s PHC reform. It is crucial to prioritize the needs of residents, overcome existing obstacles, and empower grassroots initiatives. Our findings are expected to provide practical implications for other developing countries facing similar challenges resulting from underdeveloped healthcare systems and NCD prevention and management.

## Data Availability

The raw data supporting the conclusions of this article will be made available by the authors, without undue reservation.
